# Assessing Age-Friendly Community Progress: What Have We Learned?

**DOI:** 10.1093/geroni/igab046.2140

**Published:** 2021-12-17

**Authors:** Kathy Black, Patricia Oh

**Affiliations:** 1 University of South Florida, Sarasota-Manatee, Florida, United States; 2 UMaine Center on Aging, Bangor, Maine, United States

## Abstract

The Global Network of Age-friendly Cities and Communities has grown steadily over the past decade across the United States, however surprisingly little is known regarding their accomplishments to date. We utilized content analysis to assess the progress reported by American age-friendly communities (n = 30) that joined by end of year 2015 using the Age-Friendly Community Evidence-based Tool with expanded program evaluation measures including health equity as defined by the World Health Organization. We employed deductive analytic techniques to assess reported community performance in eleven thematic areas across the range of structures and processes that characterize age-friendly efforts. We found strong evidence in the areas of leadership and governance, harnessed resources, application of age-friendly framework, and in multisector collaboration as well as reported provisions. All of the communities reported health equity aims, particularly in promoting accessible physical environments and social inclusion efforts. Our analysis further revealed areas for continued improvement.

